# Indirect prediction of graphene nanoplatelets-reinforced cementitious composites compressive strength by using machine learning approaches

**DOI:** 10.1038/s41598-024-64204-3

**Published:** 2024-06-20

**Authors:** Muhammad Fawad, Hisham Alabduljabbar, Furqan Farooq, Taoufik Najeh, Yaser Gamil, Bilal Ahmed

**Affiliations:** 1 Department of Structural Engineering, Faculty of Civil Engineering, Silesian University of Technology, Akademicka 2, 44-100 Gliwice, Poland; 2 Budapest University of Technology and Economics Hungary, Budapest, Hungary; 3 Department of Civil Engineering, College of Engineering in Al-Kharj, Prince Sattam Bin Abdulaziz University, Al-Kharj, 11942 Saudi Arabia; 4 NUST Institute of Civil Engineering (NICE), School of Civil and Environmental Engineering (SCEE), National University of Sciences and Technology (NUST), Sector H-12, Islamabad, 44000 Pakistan; 5 Western Caspian University, Baku, Azerbaijan; 6 Operation and Maintenance, Operation, Maintenance and Acoustics, Department of Civil, Environmental and Natural Resources Engineering, Luleå University of Technology, Lulea, Sweden; 7 Department of Civil Engineering, School of Engineering, Monash University Malaysia, Jalan Lagoon Selatan, 47500 Bandar Sunway, Selangor Malaysia

**Keywords:** Graphene nanoplatelets, Machine learning, SHAP analysis, Compressive strength, Prediction models, Engineering, Civil engineering

## Abstract

Graphene nanoplatelets (GrNs) emerge as promising conductive fillers to significantly enhance the electrical conductivity and strength of cementitious composites, contributing to the development of highly efficient composites and the advancement of non-destructive structural health monitoring techniques. However, the complexities involved in these nanoscale cementitious composites are markedly intricate. Conventional regression models encounter limitations in fully understanding these intricate compositions. Thus, the current study employed four machine learning (ML) methods such as decision tree (DT), categorical boosting machine (CatBoost), adaptive neuro-fuzzy inference system (ANFIS), and light gradient boosting machine (LightGBM) to establish strong prediction models for compressive strength (CS) of graphene nanoplatelets-based materials. An extensive dataset containing 172 data points was gathered from published literature for model development. The majority portion (70%) of the database was utilized for training the model while 30% was used for validating the model efficacy on unseen data. Different metrics were employed to assess the performance of the established ML models. In addition, SHapley Additve explanation (SHAP) for model interpretability. The DT, CatBoost, LightGBM, and ANFIS models exhibited excellent prediction efficacy with R-values of 0.8708, 0.9999, 0.9043, and 0.8662, respectively. While all the suggested models demonstrated acceptable accuracy in predicting compressive strength, the CatBoost model exhibited exceptional prediction efficiency. Furthermore, the SHAP analysis provided that the thickness of GrN plays a pivotal role in GrNCC, significantly influencing CS and consequently exhibiting the highest SHAP value of + 9.39. The diameter of GrN, curing age, and w/c ratio are also prominent features in estimating the strength of graphene nanoplatelets-based cementitious materials. This research underscores the efficacy of ML methods in accurately forecasting the characteristics of concrete reinforced with graphene nanoplatelets, providing a swift and economical substitute for laborious experimental procedures. It is suggested that to improve the generalization of the study, more inputs with increased datasets should be considered in future studies.

## Introduction

**Figure 1:**
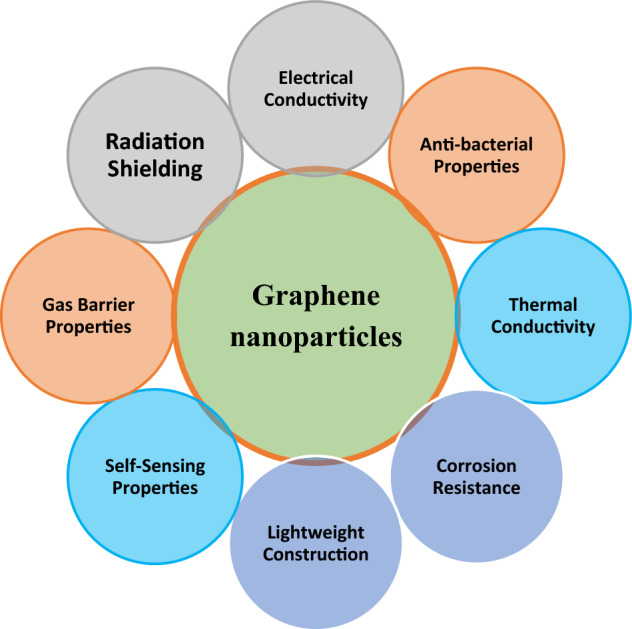
Illustration of the multifunctionality of graphene-reinforced cementitious composites^1^.

Cement-based composites are widely used in civil infrastructure due to their easy shapeability, strong compressive strength (CS), and affordability^1^. However, low tensile strength, brittleness, and vulnerability to harsh environments are significant concerns, leading to durability issues and high maintenance costs^2^. As nanotechnology (NT) advances, several attempts have been made to incorporate nanomaterials into concrete to enhance its strength and functional properties. Various nanomaterials, such as graphene nanoplatelets (GrN), nano-titanium dioxide, carbon-nanofiber (CNF), nano-silica, nano-clay, and carbon-nanotube (CNT), have been incorporated into cement composites to enhance durability, strength, and functionality^3–5^. Nanomaterials (NMs) are commonly employed to improve concrete performance by harnessing physical processes such as nucleation and filling^6–8^. In 2004, Novoselov et al.^9^ unveiled a groundbreaking two-dimensional carbon-based material called graphene, acquired through a method of exfoliation from bulk graphite. Due to its exceptional physical characteristics, including Young's modulus of 1.1 TPa and tensile strength of 130 GPa, graphene is widely regarded as the most promising carbon-based nanomaterial for diverse applications. Moreover, it boasts remarkable intrinsic features such as extraordinary thermal conductivity, a large specific surface area, and high electronic mobility^10–13^. In recent years, graphene has emerged as a standout among carbon-based nanomaterials, offering the potential to improve the performance of cement composites in multiple ways when integrated into the cement matrix^1^. The increased focus on integrating graphene into cementitious composites stems from several factors^14,15^. These include enhancing concrete's resistance to harsh environments, reducing the required amount of cement to achieve similar strength properties, preventing thermal cracking, providing fire resistance, offering excellent electrical properties for effective sensing in smart civil infrastructure, demonstrating exceptional electromagnetic interference shielding to protect humans from electromagnetic emission issues, and enabling mass production at relatively low manufacturing costs while preserving its pristine characteristics^16–23^. The multifunctionality of graphene-reinforced cementitious composites is illustrated in Fig. 1^23–25^.

In recent years, there have been continuous reports of significant improvements in the CS of concrete reinforced with graphene. The enhancement in the CS of graphene nanoplatelets-reinforced cementitious composites (GrNCC) can mainly be attributed to three factors: the nano-filler effect leading to a more compacted and refined cement matrix, the bridging effect of graphene effectively preventing and constraining the propagation of microcracks, and the large specific surface area of graphene promoting cement hydration reactions^25–27^. Wang et al.^28^ found that the flexural strength of cement paste increased by 15–24% with the addition of 0.05 wt% GrN (by weight of cement). Additionally, the CS of the GrN-cement composite increased by 3–8%. Similarly, Liu et al.^29^ reported a 14.9% enhancement in CS of GrNCC with 0.025 wt% addition of GrN. Meng and Khyat^30^ employed GrN ranging from 0 to 0.3% and observed that as the nanomaterial content, When the value rose from 0 to 0.3%, the tensile strength and energy absorption capacity exhibited respective improvements of 56% and 187%. Similarly, the flexural strength had a 59% increase, while the toughness experienced a significant boost of 276%. At the 0.2% GrN level, the UHPCs exhibited strain-hardening properties in both tension and flexure. Matalkah and Soroushian^31^ noted that the CS of the alkali-activated binder increased with the incorporation of GrN at modest concentrations. Furthermore, Li et al.^32^ investigated the utilization of surface-treated GrN in cementitious systems. Their study revealed that incorporating 0.5% GrN resulted in a 19% increase in CS. Moreover, Murugan et al.^33^ observed a 22% enhancement in CS of GrNCC with the addition of 0.02% of GrN. It is important to emphasize that the properties of concrete composites are influenced by various factors such as the kind of GrN, their concentration, and the amount of water in them. Determining the optimal GrN content in the matrix is an essential area of study in concrete composites. It is crucial to establish the association between composite qualities and numerous parameters, such as the GrN content^34–37^. Enhancing the strength characteristics of these materials is achievable by integrating the correct amount of GrN. Nevertheless, excessive or inadequate GrN content can have adverse effects on cementitious composites^38^. Carrying out mechanical tests to assess composite properties is both time-consuming and costly. These constraints make it impractical to conduct tests on numerous specimens with different GrN concentrations using empirical formulations, given the non-linear relationship between parameters. In addition, existing conventional modeling methods are inadequate in representing the complex multiscale interactions of graphene nanoparticle-reinforced composites because they lack spatial accuracy, neglect fundamental effects, and cannot account for material heterogeneity and high non-linear behaviour due to the presence of GrN. Hence, the development of ML-based predictive models for composite materials is essential to tackle these obstacles. Therefore, ML can be alternatively employed to create reliable prediction models for assessing the characteristics of GrNCC^39–41^.

Recent advancements in data science have led to the development of ML algorithms, which exhibit impressive efficacy and precision in forecasting outcomes by training predictive^42–47^. Consequently, leveraging computational modeling with data acquired from published studies presents a cost-efficient means of assessing concrete properties^48–50^. Similarly, ML-driven evaluations of concrete properties offer more cost-effective, swifter, and more flexible alternatives to traditional experimental methods^51–53^. The ML-based prediction methods consist of two main stages: training and testing. During the training phase, ML algorithms carefully analyze large datasets to reveal hidden connections and patterns within the data being analyzed^54–56^. These observed trends are used as the basis for creating a prediction model, which is then used to analyze new, unknown data during the testing phase^56–58^. The ability to deduce from existing data and process unexpected information makes ML techniques highly valuable for a diverse range of applications. To address the constraints associated with traditional modeling methodologies, the extensive utilization of ML methods is widely employed to predict the characteristics of cement-based materials. For example, Majid and Javed^58^ estimated the strength and durability characteristics of concrete by utilizing decision tree (DT) and AdaBoost regressor (AR) models. The DT and AR models exhibited exceptional precision (R = 0.99) when evaluating the characteristics of blended cement concrete. Similarly, Chen at.^59^ employed gene expression programming (GEP) to forecast the strength properties of concrete containing waste foundry sand. The GEP approach exhibited excellent precision(R = 0.99) in estimating the output. Biswas et al.^60^ utilized a support vector machine (SVM) to calculate the level of carbonation of concrete incorporating fly ash. The model was trained and validated using a dataset of 300 data points collected from prior research. The results showed a strong correlation (R  > 0.95). Amin et al.^61^ utilized extreme gradient boosting (XGBoost), random forest (RF), and light gradient boosting machine (LightGBM) approaches to examine the bond Strength of fiber-reinforced polymer with concrete. The LightGBM model exhibited greater precision than the RF and XGBoost approaches. Moreover, Nafees et al.^62^ used GEP and adaptive neural fuzzy detection systems (ANFIS) to forecast the CS and tensile strength of concrete containing silica fume. The GEP and ANFIS models provided higher prediction accuracy (R  > 0.98) to estimate the strength properties of concrete containing silica fume. Wu and Huang^63^ forecasted the strength of concrete using a categorical boosting (CatBoost) approach optimized honey badger algorithm. The CatBoost approach showed extremely accurate predictions for CS and flexural strength of concrete.

Although there is an extensive study that forecasts the properties of different cementitious composites, there have been few studies that particularly examine the use of ML to calculate the properties of GrNCC. Dong et al.^64^ utilized the XGBoost model to forecast the CS of GNPC, achieving a strong R-value of 0.95. Montazerian et al.^65^ employed DT and SVM to predict the strength of concrete, including graphene derivatives. These ML methods exhibited robust prediction accuracy. Likewise, Sun et al.^66^ utilized Random Forest (RF) to create an ML model for the CS of concrete incorporating graphite and slag powder. The RF model exhibited exceptional predictive accuracy, proven by a high R-value of 0.99. Moreover, Yang et al.^67^ developed several models, such as AutoGluon-tabular (AGT), SVR, RF, and ANN. Among these models, the ANN demonstrated superior accuracy compared to the other models. Although the aforementioned models attained high accuracy in forecasting the properties of GNPC, the aspect of interpretability has frequently been overlooked despite its importance in building confidence in ML models for real-world applications. Therefore, to overcome the issues in the previous models, this study utilizes different ML methods to develop prediction models for the CS of GrNCC. The primary objectives of the present study are (1) to establish robust and reliable ML-based prediction models using DT, CatBoost, ANFIS, and LightGBM, (2) to conduct a comparative study of the developed ML models, (3) to utilize SHAP analysis to unveil the controlling parameters in assessing the CS of GrNCC.

## Research methodology

### Data collection

A comprehensive literature review was conducted to gather experimental results regarding the CS of GrNCC. Relevant data from a variety of published articles^16,17,26,28,29,68–93^ that provide thorough insights into the factors and characteristics that affect compressive strength was compiled, resulting in a database comprising 172 data points as depicted in Appendix A. The selective method of data compilation looked to enhance the dataset with high-quality and appropriate information to conduct a thorough analysis of factors influencing compressive strength.

### Selection of input variables

An extensive literature review was carried out to examine the factors influencing the CS GrNCC. Key factors identified through this review were compiled and utilized to define the input characteristics of the datasets. In this study, the inputs were chosen based on the literature studies^94,95^. The final selected inputs are classified into three categories: raw materials properties, GrNs suspension method, and testing age. The raw materials include sand content (SC), and GrN properties such as GrN diameter (GD), GrN thickness (GT), and GrN content (GC). Moreover, the GrN dispersion method includes ultrasonication (US), and curing age (CA). The output property is the compressive strength (CS) of GrNCC. GrNCC specimen preparation typically follows the procedures outlined in ASTM C39^96^. Consequently, the composition of cement in the acquired data record was standardized to 1, with the dosage values of other additives adjusted to percentages relative to the mass of the cement. Physical parameters such as GrN thickness, content, and diameter were considered, as they impact the compressive strength of GrNCC. However, the aspect ratio was not taken into account, as it represents the ratio of diameter to thickness. Based on collected data points, fine sand over medium sand was the preferred choice among researchers studying GrNCC. Additionally, graphene oxide (GrO) was more commonly used than reduced graphene oxide (rGrO) in GrNCC studies. This study specifically employs GrO, a type of graphene nanoparticle, to construct the database. Chamber curing was favoured over water curing in approximately twice as many studies. Furthermore, the inclusion of a superplasticizer in the model was omitted, which is consistent with recommendations found in the literature^97^. The duration of ultrasonication processing emerged as a critical factor in assessing the dispersion impact of graphene nanoparticles in water, denoted by a parameter termed US, with values input in hours. Regarding curing conditions, curing age was identified as a pivotal parameter regulating the hydration status of GrNCC, exerting a significant influence on the attributes under investigation. Theoretically, compressive strength is considered to be unaffected by sample size^98^. Therefore, it was deemed suitable to exclude specimen dimensions as an input variable. Finally, considering the thorough assessment of the literature as discussed earlier, the seven most significant inputs were chosen for the final database development. These factors are well-documented and widely recognized for their influence on compressive strength. The descriptive statistics of the collected dataset of these seven inputs are provided in Table 1.

**Table 1:** Statistical analysis of the acquired dataset.

Statistical parameter	SC	GT (nm)	GC (wt%)	GD (µm)	US (hr)	w/c	CA (days)	CS (MPa)
Mean	1.37	3.39	0.26	3.38	0.49	0.40	19.70	48.51
Sample variance	2.24	27.01	0.89	47.21	0.68	0.012	117.16	365.18
Range	3	26.9	6.39	49.92	3	0.52	27	79.67
Standard deviation	1.49	5.19	0.94	6.87	0.82	0.11	10.82	19.10
Mode	0	1	0.03	0.55	0	0.37	28	53.32
Skewness	0.16	2.63	2.44	1.60	2.37	1.24	− 0.62	0.20
Kurtosis	− 1.99	8.18	7.12	6.55	4.66	1.43	− 1.49	− 0.60
Maximum	3	27.6	6.4	50	3	0.72	28	94.26
Minimum	0	0.7	0.01	0.07	0	0.2	1	14.59
Normalized range	1	4.2	0.13	1.81	4.17	0.17	0.96	0.85

**Figure 2:**
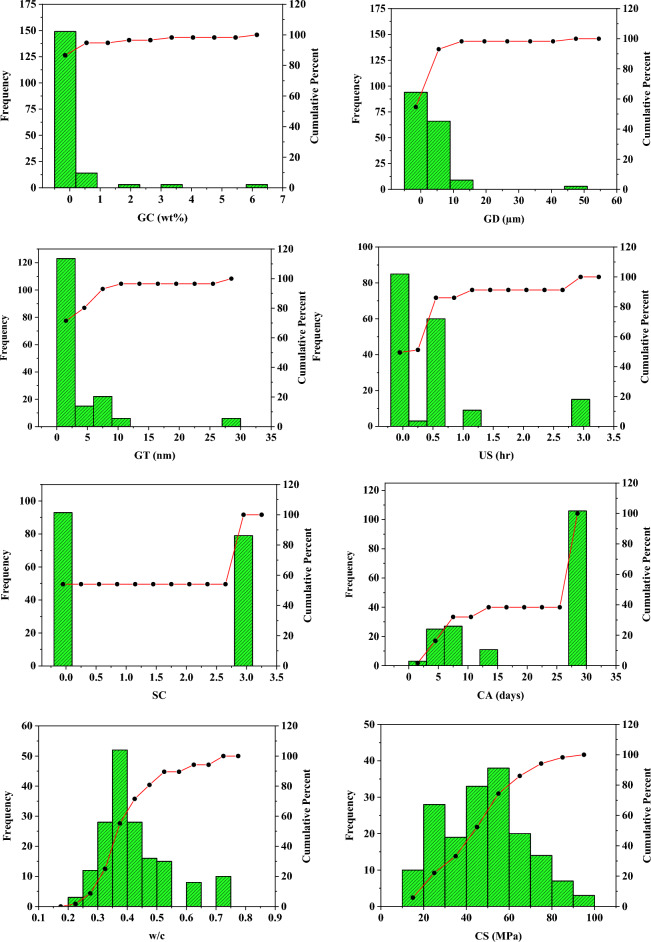
Frequency distribution histogram of the parameters.

Statistical analysis for input variables, including measures of data distribution, central tendency (mean, median), extremes, and trends, enhances the comprehension of the database, thereby improving its overall understanding^59,99,100^. It is suggested that the kurtosis and skewness of the database must be within ± 3 and ± 10, respectively. These parameters are within the recommended range for the collected dataset. In addition, the CS of the GrNCC ranges from 14.59 MPa to 94.26 MPa. This statistical analysis showcases the adaptability of the constructed machine learning models to a wider range of data, thus enhancing their practical utility. Furthermore, the variable's frequency distribution histograms are provided in Fig. 2.

### Preprocessing of the data

The preprocessing conducted on the original datasets is crucial for enhancing the outcomes and performance of the machine learning (ML) modelling process. During the preprocessing stage, outliers were identified and subsequently removed from the final database to enhance the overall integrity and reliability of the dataset. The outliers were addressed by employing a mutation procedure, in which irregular data points were replaced with the respective mean values. To avoid human bias in the model evaluation, the original datasets were split into training and testing sets at a ratio of 7:3. Therefore, 70% of data was employed for training (learning) the model, while 30% of data was utilized for testing the developed model.

#### Correlation of parameters

**Figure 3:**
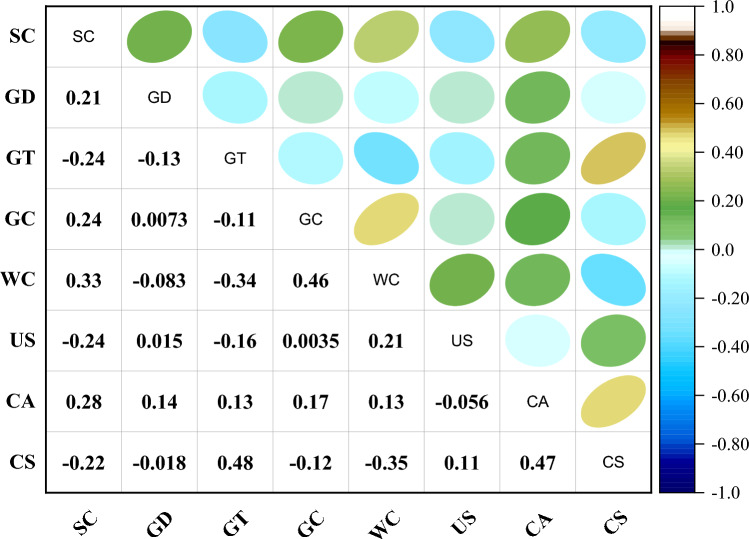
Correlation heatmap of the dataset.

Prior to additional processing, it is essential to explore relationships between input parameters. Pearson's correlation coefficient (r), typically ranging from − 1 to 1, is commonly used to measure linear correlation between any two parameters^101^. As the absolute value of the r decreases, the correlation between two parameters also decreases, and vice versa. Smith^102^ suggests that a r exceeding 0.80 indicates a strong relationship between two parameters. This type of issue is often referred to as the multi-collinearity problem^98^. To avoid multi-collinearity problems in the acquired dataset, it is essential that the r must be lower than 0.8 between any two parameters^43,59,99–104^. It is evident that there is no risk of multi-collinearity as all R-values, whether negative or positive, are below 0.8, as shown in Fig. 3. Moreover, GrN thickness (+ 0.48) and curing age (+ 0.47) have the highest positive correlation with CS, while the water-to-cement ratio has the highest negative correlation (− 0.35) with CS.

#### Normalization of data

Variations in the scales and ranges of inputs can affect the performance of ML models^105^. As indicated in Table 1, both input and output parameters exhibit wide ranges. For example, GT ranges from 0.7 to 27.6, GD varies from 0.072 to 50, and CS ranges from 14.59 to 94.26. To mitigate potential bias stemming from these variations, the original database underwent normalization before being utilized for ML modeling^106^. As a component of the data preprocessing for the ML models, the StandardScaler procedure was utilized. Its purpose is to normalize the functional scale of the data record^107,108^. This involves eliminating the average and clearing each parameter to have a unit difference (variation)^109^. StandardScaler ensures uniform scaling across various parameters, thereby reducing potential biases in the data and improving the overall efficacy of the models^53^. The exact normalized range can be seen in Table 1.

### Utilized ML approaches

When it comes to tangible substances, the benefits of using machine learning techniques are very noticeable. Conventional statistical methods frequently face difficulties in capturing the complex, non-linear connections between input factors and material qualities that are inherent in concrete compositions. Machine learning methods, such as decision trees, light gradient boosting, catboost, and adaptive neuro-fuzzy inference systems (ANFIS), provide a strong and reliable solution to this problem. These methods are highly proficient at analyzing intricate datasets, enabling the detection of nuanced patterns and correlations that may be overlooked by traditional approaches. Our study improves the accuracy of predicting concrete material qualities by utilizing machine learning skills. This, in turn, allows for more exact design and optimization processes. Furthermore, the knowledge obtained from machine learning analyses enhances comprehension of the fundamental principles that control specific actions, enabling researchers and professionals to make well-informed choices in the field of material design and engineering applications.

#### Decision tree (DT)

**Figure 4:**
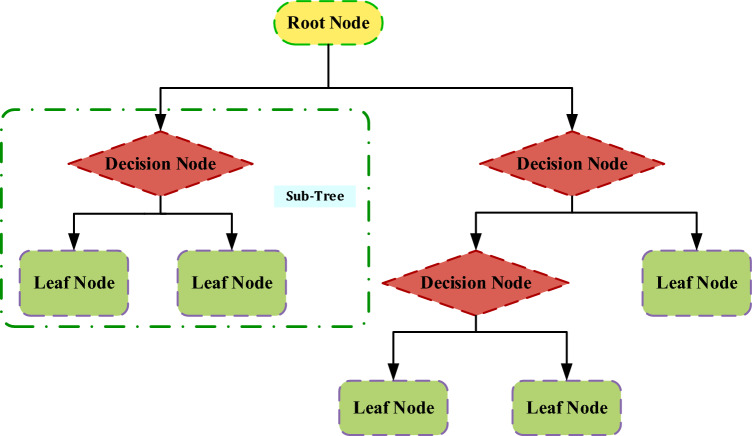
Architecture of DT.

The Decision Tree (DT) algorithm is a widely used ML approach employed for both classification and regression tasks. The DT algorithm operates by iteratively dividing the data into smaller groups according to the values of input attributes^110–112^. At each iteration, the algorithm chooses the feature that optimally divides the data into subsets with the highest level of purity, often utilizing measures such as Gini impurity or entropy. This process persists until a specified stopping requirement is satisfied, such as attaining the maximum depth of the tree or encountering nodes with a minimal number of samples, as depicted in Fig. 4^112,113^. In order to create predictions, the algorithm navigates around the tree, starting from the root node and ending at a leaf node. At the leaf node, it assigns the most common class for classification tasks or the average value for regression tasks. Decision trees are easily understandable, but they are susceptible to overfitting. This issue can be mitigated by employing techniques such as pruning or ensemble methods like RF and other boosting methods^114^.

#### LightGBM

LightGBM is a gradient-boosting framework that is widely used in ML tasks for classification, regression, and ranking. LightGBM sequentially builds DTs, with each new tree focusing on the mistakes of the previous ones^115,116^. Unlike traditional gradient boosting methods, LightGBM uses an innovative method called gradient-based one-sided sampling to construct trees, which notably speeds up the training procedure and reduces memory usage. Additionally, it implements leaf-wise tree growth, where it grows the tree node-by-node, selecting the leaf with the maximum delta loss, heading to faster convergence^47^. During prediction, LightGBM traverses the ensemble of trees and calculates the final prediction by summing up the predictions from each tree. Its efficient implementation and ability to handle large datasets make LightGBM a popular choice for various machine-learning applications^117,118^.

#### CatBoost

CatBoost, a gradient-boosting framework, is particularly adept at handling categorical features effectively^119^. Unlike other frameworks, CatBoost integrates predictions from various models to produce a more stable and generalizable output. This approach helps prevent overfitting by averaging errors across models while maintaining high prediction performance. CatBoost employs symmetric trees, ensuring consistent splitting conditions across nodes at the same depth, which aids faster computation and helps control overfitting^45^. Its splitting method employs a strategy, selecting the split with the least penalty, which optimizes decision-making. Boosting in CatBoost leverages categorical information to improve model generalization, enhancing data robustness. Notably, CatBoost provides specialized support for categorical features through features like one_hot_max_size and cat_features, facilitating faster training and addressing overfitting concerns. In summary, CatBoost offers a comprehensive solution for ML tasks, especially in scenarios with diverse data types and the need to mitigate overfitting risks^120^.

#### ANFIS

**Figure 5:**
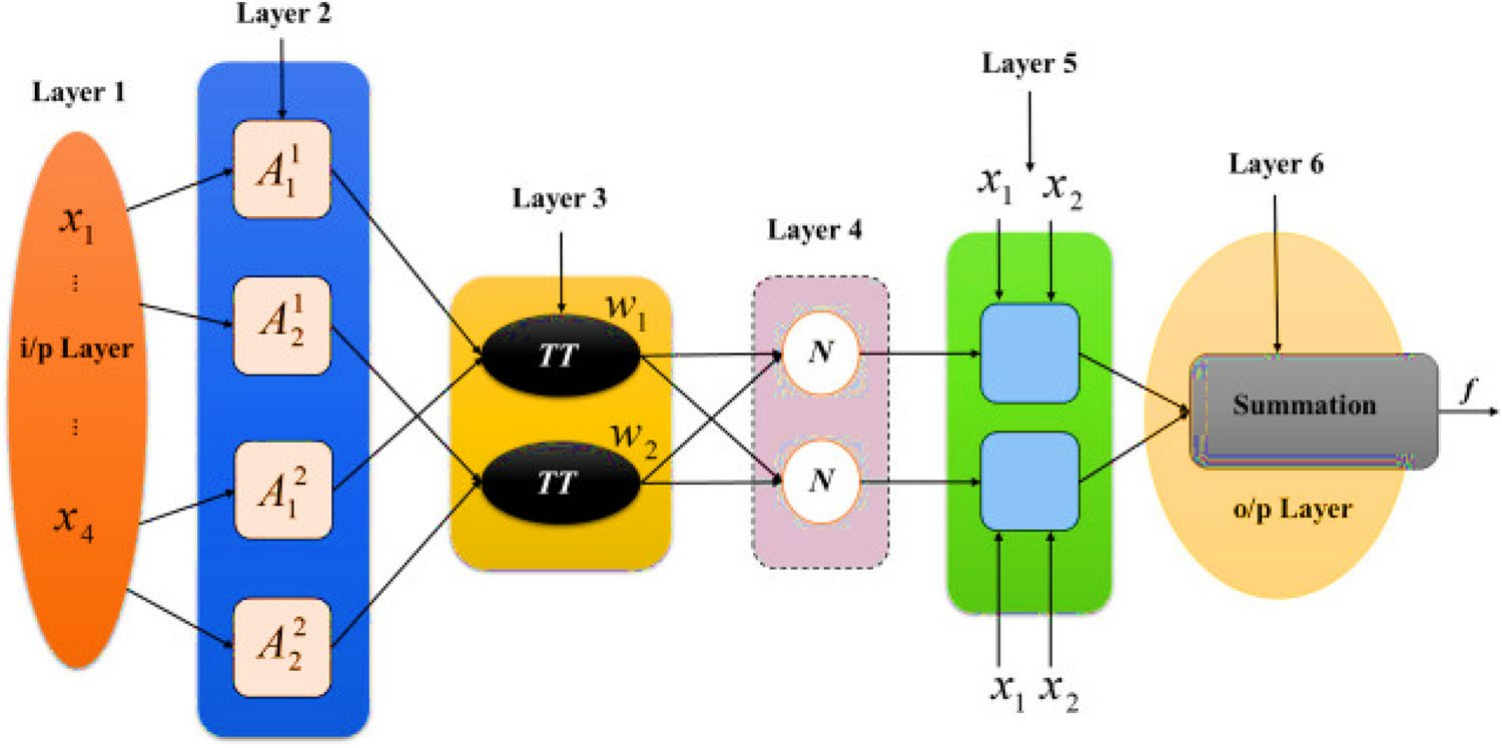
Architecture of ANFIS.

Jang^121^ introduced the ANFIS approach that integrates artificial neural networks with fuzzy logic. ANFIS, is a hybrid computational model that combines the adaptive capabilities of neural networks with the human-like reasoning of fuzzy logic. It aims to create a flexible and interpretable framework for solving complex problems, particularly in pattern recognition, system identification, and control applications. ANFIS employs a structure consisting of five layers: the input layer (GrNCC ingredients), fuzzification layer, rule layer, defuzzification layer, and output layer^122–125^. In ANFIS, the input layer collects input data, which is then passed to the fuzzification layer, where linguistic variables are defined to represent the input data's characteristics. The rule layer finds out the firing intensity of each rule based on the input variables' membership functions and fuzzy rules^126^. These rules capture the relationship between input and output variables using if–then statements, which are derived from expert knowledge or data-driven learning. Next, the defuzzification layer aggregates the outputs of each rule to generate a crisp output value. This layer typically utilizes methods like weighted averaging or centroid defuzzification to combine the fuzzy outputs^127^. Finally, the output layer (CS) creates the overall output of the ANFIS model. Furthermore, ANFIS employs a hybrid learning algorithm to adjust its parameters and optimize its performance. This algorithm combines gradient descent with least squares estimation to iteratively update the parameters of the model based on the training data. By adaptively adjusting its parameters, ANFIS can learn complicated non-linear links between input and output features^128,129^. The architecture of ANFIS is shown in Fig. 5^130^.

### Parameter optimization

Hyperparameter optimization is an essential step in improving the efficacy of the ML models. In this study, an iterative process (trial and error) was used to maximize the models' prediction power by fine-tuning a combination of parameters and proposing the best combination. The performance indicators as discussed below, were used as a stopping criterion to identify the best parameters. The ideal parametric setting utilized for all developed models is shown in Table 2.

**Table 2:** Parametric optimization.

Algorithm	Hyperparameter	Explanation	Optimal Value
DT	Min. number of instances in leaves	The minimum number of samples required for the splitting of nodes	2
	Do not split subsets smaller than	Prohibits the algorithm from splitting nodes with fewer instances than the specified number	5
	Limit the maximal tree depth	Constrains the depth of the classification tree to the specified number of node levels	100
LightGBM	Number of estimators	The number of trees within the forest ensemble	100
	Learning rate	It signifies the degree to which newly acquired information takes precedence over existing information	2
CatBoost	Number of estimators	The number of trees within the forest ensemble	100
	Learning rate	It signifies the degree to which newly acquired information takes precedence over existing information	2
ANFIS	No of Nodes	Quantity of nodes present in the structure	10
	Epochs	The designated number of epochs for the training process	50
	Linear parameters	Count of linear parameters influencing the model's behaviour	70
	Fuzzy	Count of fuzzy rules governing the system	8
	Non-linear parameters	Total number of non-linear parameters influencing the model	66
	MFs	Number of membership functions (MFs) utilized in the system	20
	Error	The desired error goal was set during the training phase	0

### Performance metrics

Several performance metrics such as mean absolute error (MAE), correlation coefficient (R), mean square error (RMSE), and a10-index, were employed to assess the efficacy of the developed models. The expression of these metrics is given as Eqs. 1–4.

1 $$RMSE = \sqrt {\frac{{\sum\limits_{{i = 1}}^{n} {\left( {{\varvec{Ac}}_{i} - {\varvec{Pr}}_{i} } \right)^{2} } }}{n}}$$

2 $${\text{MAE}} = \frac{{\sum\nolimits_{{{\mathrm{i}} = 1}}^{{\mathrm{n}}} {\left| {{\varvec{Ac}}_{{\mathrm{i}}} - {\varvec{Pr}}_{{\mathrm{i}}} } \right|} }}{{\text{n}}}$$

3 $${\text{R}} = \frac{{\sum\nolimits_{{{\mathrm{i}} = 1}}^{{\mathrm{n}}} {\left( {{\textbf{Ac}}_{{\mathrm{i}}} - \overline{{{\textbf{Ac}}}} _{i} } \right)\left( {{\textbf{Pr}}_{{\mathrm{i}}} - \overline{{{\textbf{Pr}}}} _{i} } \right)} }}{{\sqrt {\sum\nolimits_{{{\mathrm{i}} = 1}}^{{\mathrm{n}}} {\left( {{\textbf{Ac}}_{{\mathrm{i}}} - \overline{{{\textbf{Ac}}}} _{i} } \right)^{2} } \sum\nolimits_{{{\mathrm{i}} = 1}}^{{\mathrm{n}}} {\left( {{\textbf{Pr}}_{{\mathrm{i}}} - \overline{{{\textbf{Pr}}}} _{i} } \right)^{2} } } }}$$

4 $${\text{a}}10-{\text{index}}= \frac{{\mathrm{N}}{10}}{\mathrm{n}}$$

where ${\textbf{Pr}}_{\mathrm{i}}$ and ${\textbf{Ac}}_{\mathrm{i}}$ show the anticipated and actual values, respectively, while $\overline{\textbf{Pr}}_{\mathrm{i}}$, and $\overline{\textbf{Ac}}_{\mathrm{i}}$ denote the mean anticipated and actual values, respectively, and n denotes the number of data points in the dataset. In the model calibration, the optimal model is identified by minimizing errors such as MAE and RMSE while also achieving a high R. Researchers suggest that a strong correlation, ideally surpassing 0.8, indicates robustness, with a perfect correlation being represented by an R-value of 1. Additionally, the N10 metric signifies the count of predictions-to-actual ratio falling within a range of 0.90–1.10. A higher a10-index value, nearer to one, indicates superior model performance^51,131^.

### Shapley additive explanation (SHAP) interpretability

In the current study, the SHAP method was employed for model interpretability. SHAP stands out as an efficient tool for elucidating complex ML models, offering a transparent and explainable approach^132^. Based on cooperative game theory, SHAP values are a unique way to figure out feature importance by giving each predictor's role in model predictions a value. SHAP helps us understand the factors that affect model outputs better by carefully measuring the effects of each feature. This approach not only helps improve models, but it also encourages transparency and confidence, especially when important decisions need to be made^133^. Through equitable allocation of feature contributions, SHAP enables a nuanced grasp of how certain variables influence estimates, thereby advancing clarity and accountability in the intricate realm of ML models^134^.

## Results and discussion

### Regression slope analysis

**Figure 6:**
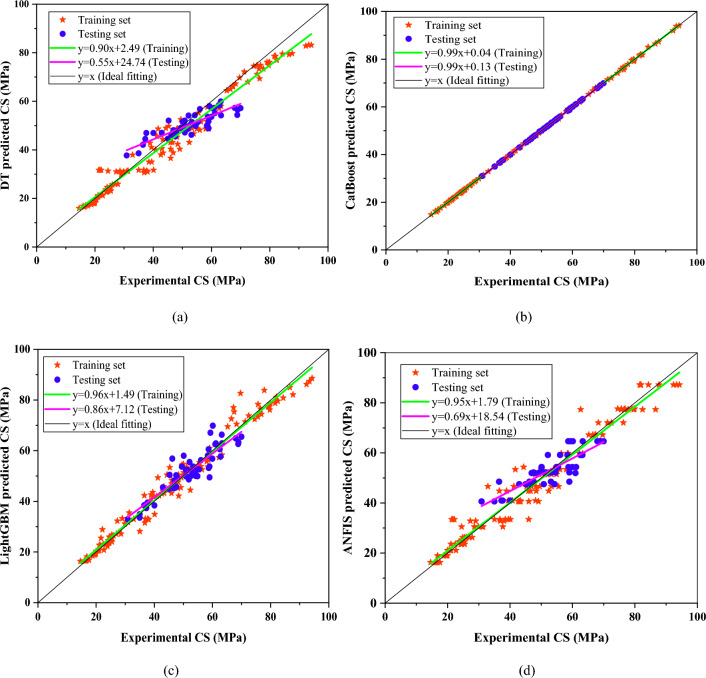
Regression plots of the models: (**a**) DT, (**b**) CatBoost, (**c**) LightGBM, (**d**) ANFIS.

Regression slope is commonly utilized to assess the effectiveness of machine learning models. In a regression plot, actual values are depicted on the horizontal axis, while the model's predicted values are shown on the vertical axis, as depicted in Fig. 6. A regression slope approaching one (> 0.8) typically indicates that the model's predictions closely align with the observed values. In training, the DT model demonstrated a slope of 0.90, showcasing a strong alignment between predicted and actual values. However, during testing, the slope dropped to 0.55, notably falling below the expected threshold of 0.80. Furthermore, the CatBoost model exhibited a higher slope of 0.99 in both phases, indicating its robust performance in estimating the CS. Similarly, the LightGBM model showed 0.96 and 0.86 for training and testing, respectively. On the other hand, the ANFIS model showed a higher slope (0.95) in training; however, it demonstrated a lower slope in testing (0.69). Overall, the CatBoost and LightGBM models provided higher slopes in both sets, indicating their higher prediction precision in estimating the out. In addition, the fitting lines of testing and training for these two models are in close proximity to the ideal fit line.

### Statistical analysis

The statistical evaluation of the established models is given in Table 3. Several statistical metrics, such as R, a-10 index, MAE, and RMSE, are employed to comprehensively assess the efficacy of the models. The R-values in training are 0.9835, 0.9999, 0.9665, and 0.9768 for DT, CatBoost, LightGBM, and ANFIS models, respectively, while for testing, are 0.8708, 0.9999, 0.9043, and 0.8663, respectively. The correlation (R) of all the models is higher than the recommended value of 0.80, indicating the models' robust predictive performance. However, the CatBoost model provided significantly more than 0.80 (closer to 1), indicating the higher efficacy of the CatBoost model in estimating the CS of GrNCC. Furthermore, the lowest MAE _training_ was observed for the CatBoost model (0.1405), while the highest MAE _testing_ was noted for the DT model (3.5245). A similar observation was also noted in the testing phase. The DT model exhibited the lowest a10-index values for both training and testing sets, indicating a significant deviation of predictions from the actual values, surpassing the 10% error range in the majority of cases. Conversely, the CatBoost model showcased the highest a10-index values of 0.9918 for training and a perfect score of 1.00 for testing. This signifies that the CatBoost model consistently maintained a high level of accuracy, with the majority of its predictions falling within the 10% error margin of the true values. A higher TA value of a10-index is also observed for the ANFIS model, as shown in Table 3.

**Table 3:** Performance evaluation of the models.

Model	R	MAE (MPa)	RMSE (MPa)	a10-index
Training				
DT	0.9835	3.5245	4.6152	0.6967
CatBoost	0.9999	0.1405	0.2234	0.9918
LightGBM	0.9865	2.5493	3.5311	0.8197
ANFIS	0.9768	3.2532	4.6270	0.9918
NLR	0.74	5.45	6.34	0.63
Testing				
DT	0.8708	4.6242	5.8580	0.6471
CatBoost	0.9999	0.0704	0.0979	1.0000
LightGBM	0.9043	3.1017	4.0340	0.8627
ANFIS	0.8662	4.0400	4.8188	1.0000
NLR	0.69	6.45	6.84	0.6

### Comparative analysis of the models

**Figure 7:**
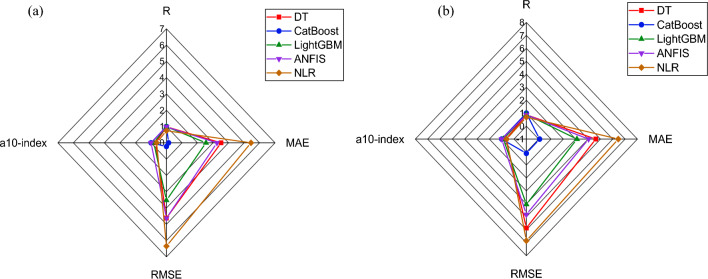
Spider plots: (**a**) Training, (**b**) Testing.

**Figure 8:**
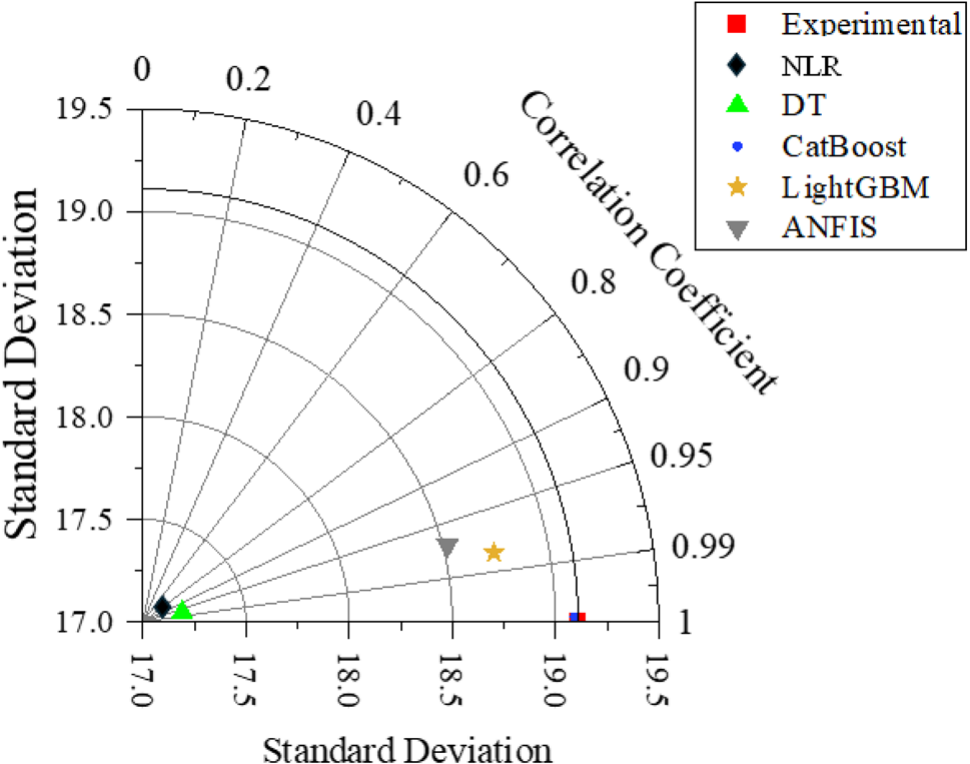
Taylor diagram.

The established are compared to select the best prediction models for forecasting the strength of GrNCC, as illustrated in Fig. 7. The CatBoost model showed the highest correlation (R) and lowest error (MAE, RMSE). The CatBoost model outperforms the DT model by 96.01% in _training_, surpasses the LightGBM model by 94.48%, and exceeds the ANFIS model by 95.68%. Similarly, the MAE_testing_ of the CatBoost model is significantly lower than that of the other developed models, indicating the robust prediction performance of the CatBoost model in forecasting the output. The validation of the models' comparative analysis is reinforced through the Taylor diagram, depicted in Fig. 8. A Taylor diagram offers a comprehensive view of the prediction performance across multiple models. It allows for a concise comparison of correlation coefficients, standard deviations, and variance ratios, highlighting the strengths and weaknesses of each model in capturing the observed variability. Plotting these metrics on a single diagram, facilitates the identification of models that exhibit superior predictive skill and provides valuable insights for model selection. The optimal model is distinguished by its proximity to the benchmark (highlighted in red to represent experimental data). The model that exhibits the closest alignment with the benchmark is identified as the best-performing model. It can be observed that the CatBoost model is the closest to the benchmark symbol, followed by LightGBM, ANFIS, and the DT model. Hence, the models can be rated based of their accurateness as follows: CatBoost, LightGBM, ANFIS, and DT. Finally, previous studies have highly recommended comparing the efficacy of the ML models with non-linear regression (NLR) models. Therefore, the current study also used NLR as a benchmark to assess the performance of the models. It is obvious from Figs. 7 and 8 that the proposed models surpassed the NLR models with higher R^2^ values. Further, to provide additional insight into the comparative analyses, the estimated equations derived from linear regression (LR) models are presented. Equation (5) depicts the essence of the model, offering a concise representation of the relationships established during the training process while estimating the compressive strength of GrNCC. This equation can assist as a valuable tool for understanding the underlying dynamics and further contributes to the comprehensive evaluation of the model's predictive capabilities. Overall, comparative analysis shows that the suggested ML models can be employed to evaluate the compressive strength of the GrNCC.

5 $${\text{CS (MPa)}} =49.59-1.4{\text{SC}}-0.16{\text{GD}}+1.13{\text{GT}}+0.07{\text{GC}}-57.89{\text{WC}}+5.53{\text{US}}+2.93{\text{CA}}$$

### Comparison with literature models

A comparative analysis between the models proposed in this study and those established in existing literature for calculating the CS of GrNCC is listed in Table 4. Alabduljabbar et al.^135^ utilized the GEP model to anticipate the strength of GrNCC. It has been reported that the GEP model demonstrated robust performance as compared to traditional statistical methods. The GEP approach depicts significant R^2^-value of 0.92. Additionally, Yang et al.^67^ formulated multiple models, including RF, SVR, AutoGluon-tabular (AGT), and ANN. Among these, the ANN model exhibited exceptional accuracy in estimation. Montazerian et al.^65^ employed three different ML algorithms, specifically SVR, DT, and MLP, to forecast the strength of cementitious materials incorporated with GrNs. These models exhibited similar levels of prediction accuracy. Furthermore, Khan et al.^136^ utilized DT, AR, and BR models for forecasting the strength of GrNCC. The DT and AR models provided similar performance when estimating the output. Sun et al.^66^ established predictive model for the CS of cementitious composites containing nano materials. The author employed random forest (RF) method enhanced with beetle antennae search (BAS) for optimization, and revealed that RF-BAS model demonstrated outstanding predictive accuracy. Dong et al.^137^ applied a Bayesian-tuned XGBoost model to forecast the CS of GrNCC. The author revealed that Bayesian model achieved a high correlation coefficient (R^2^) of 0.947 with less mean absolute error of 3.44, as detailed in Table 4. In addition, CatBoost, decision tree, LightGBM, and ANFIS is employed in this study. CatBoost model demonstrated significantly lower error and higher accuracy compared to the existing literature models, showcasing superior performance and outperforming them comprehensively as illustrated in Table 4.

**Table 4:** Comparison with literature models.

Ref	Model	Interpretability	Performance	
			MAE	R
^98^	GEP	SHAP	1.62	0.96
	MLR		11.22	0.62
	NLR		7.70	0.73
^66^	RF-BAS	Parametric analysis	–	0.99
^64^	Bayesian-tuned XGBoost	–	3.44	0.94
^67^	SVR	Feature importance of using AGT	5.46	0.90
	RF	SHAP	4.93	0.91
	AGT		3.69	0.96
	ANN-I		3.75	0.95
	ANN-II		3.77	0.95
	ANN-III		3.54	0.95
	DT		3.53	0.98
^44^	AR		3.45	0.98
	BR		4.87	0.95
This study	CatBoost	SHAP	0.0704	0.9999

### SHAP interpretability

**Figure 9:**
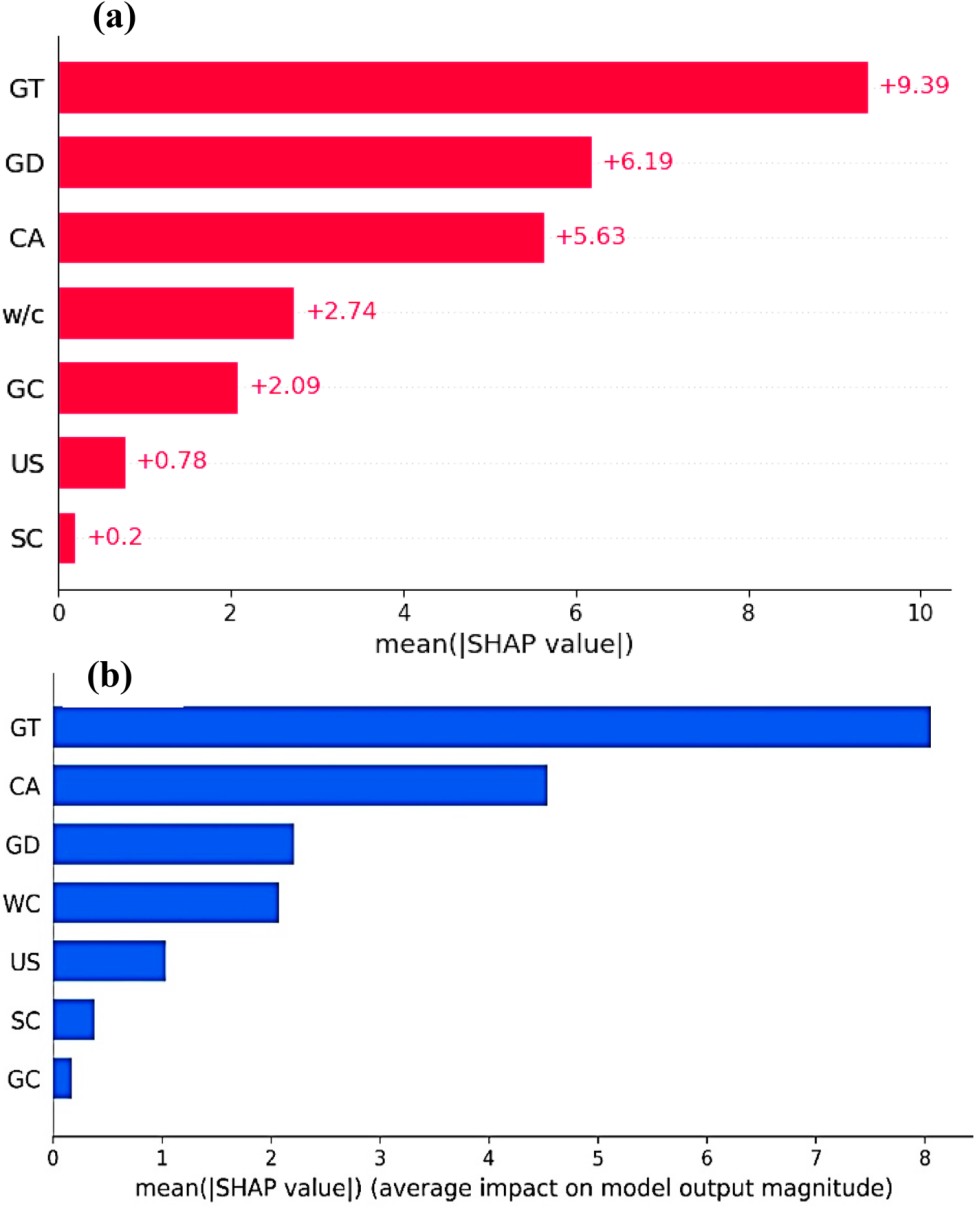
SHAP features importance plots.

**Figure 10:**
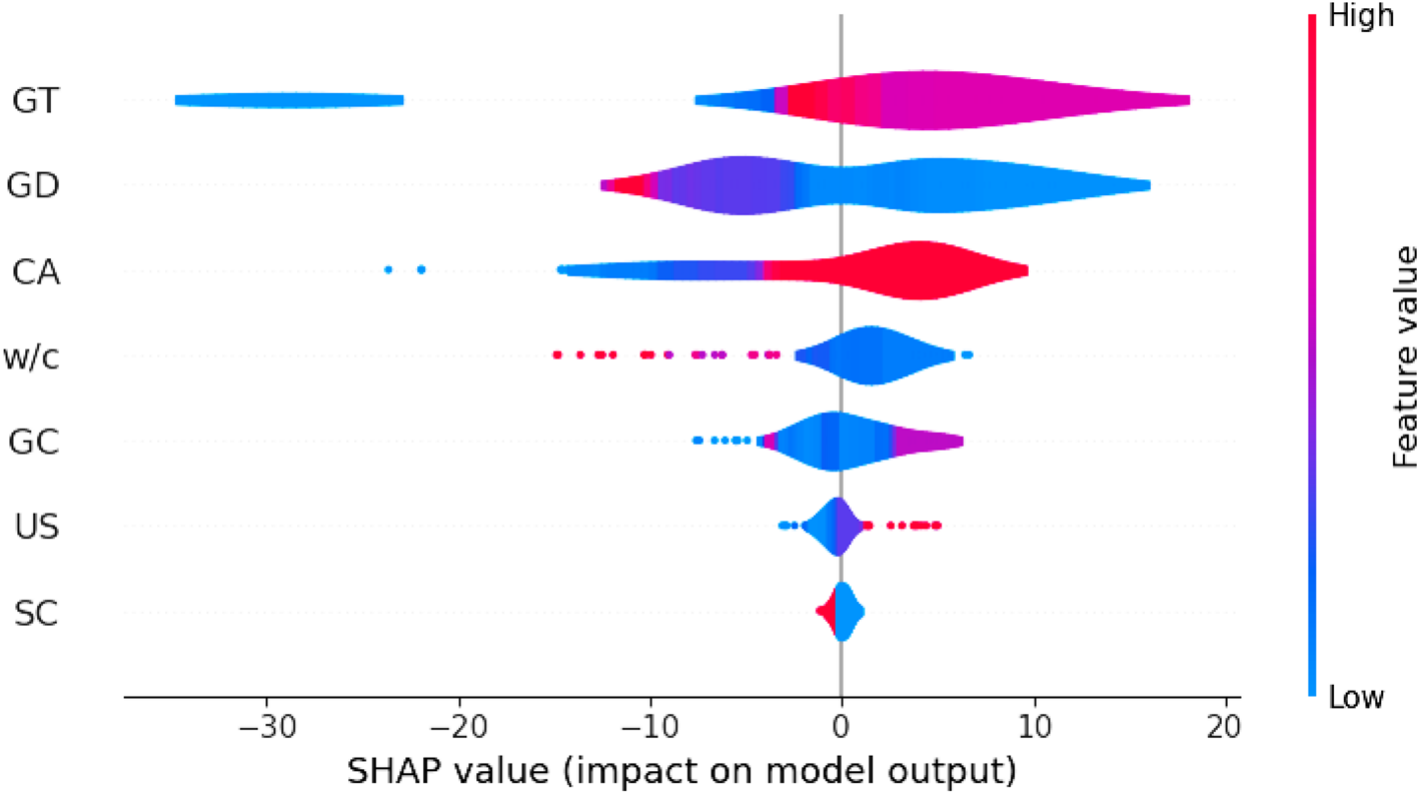
SHAP summary plot.

Generally, ML models are often regarded as black boxes, lacking transparency. Therefore, the SHAP interpretability method was selected for model interpretability^135,138^. In this study, the best prediction model (CatBoost model) was chosen for SHAP analysis. Figure 9a illustrates the SHAP feature importance plot of inputs. The thickness of GrN plays a pivotal role in GrNCC, significantly influencing CS and consequently exhibiting the highest SHAP value of + 9.39 for CS. The diameter of GrN, curing age, and w/c ratio are also prominent features in assessing the CS of GrNCC, as indicated by their higher SHAP values of + 6.19, + 5.63, and + 2.74, respectively. The remaining feature has the least significant SHAP values. While Fig. 9b provides an overview of the significance of various features, it does not reveal whether these features correlate positively or negatively with the outputs. Hence, a SHAP summary plot, as depicted in Fig. 10, is provided to illustrate the correlation between parameters and CS. Every data point on the summary plot has specific information about a Shapley value that corresponds to the features. By utilizing SHAP analysis, it is possible to compare the global mean with the model's output to identify individual causes, and features are then ordered according to their significance. In addition, the x-axis represents the negative (left) and positive (right) impacts of features, illustrating their influence on both extremes. Feature values are represented by the x-axis and y-axis in the model, emphasizing their significance and influence. For instance, a higher level of GT is linked with a higher SHAP value of CS, indicating that the higher GT positively impacts the CS. Similarly, the higher curing age also positively impacts the CS of GrNCC. In contrast, higher w/c and GD negatively impact the CS of GrNCC. Furthermore, based on the SHAP analysis, the US and SC have less considerable influence on the CS. The findings of the study are in close agreement with the literature^44^.

### Graphical user interface

**Figure 11:**
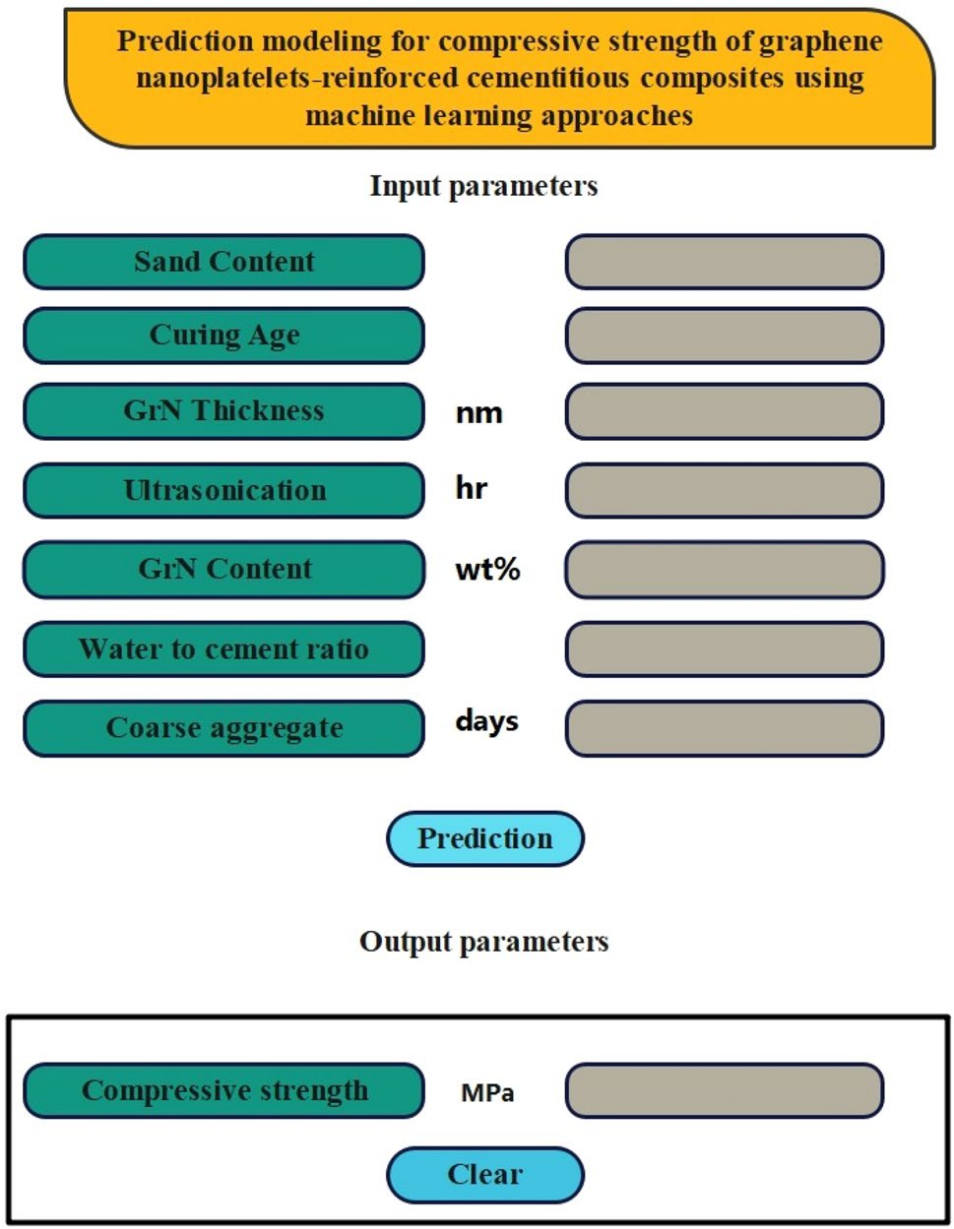
Graphical user interface of nano composite material.

This section presents the establishment of a user-friendly graphical user interface (GUI), as shown in Fig. 11. The graphical user interface (GUI) consists of a toolbar, an input area, and an output area. Researchers can easily input the seven variables into the designated input area. By selecting the "Predict" button in the output section, they can retrieve the expected values of GrNCC. The input portion has seven parameters that exert an influence on the strength of nano material concrete. This graphical user interface (GUI) can be utilized by engineers to generate concrete that fulfills the specifications and demand requires to construct building. The graphical user interface (GUI) was specifically created to prioritize user-friendliness, enabling researchers to rapidly study and forecast the behavior of nano material-CS. The authors aim to empower researchers to fully utilize the possibilities of the proposed model in their research by developing this GUI.

## Conclusion

This research utilized innovative ML models to estimate the CS of GrNCC. To construct the database for model development, 172 data points of CS were gathered from published experimental studies. The efficacy and performance of these models were assessed using diverse statistical metrics. Moreover, the SHAP explanatory technique was employed to interpret the models. The major findings of the study are as follows:

1. The suggested DT, CatBoost, LightGBM, and ANFIS models exhibited excellent prediction efficacy with R-values of 0.8708, 0.9999, 0.9043, and 0.8662, respectively. Similarly, the MAE score of these established models is 4.6242, 0.0704, 3.1017, and 4.0400, respectively. The developed models exhibited a close alignment with the actual CS values, underscoring their efficacy in accurately calculating the CS.
2. While all the suggested models demonstrated higher accuracy in predicting compressive strength, the CatBoost model exhibited exceptional prediction efficiency. The CatBoost model showed the highest correlation (R) and lowest error (MAE, RMSE). The CatBoost model outperforms the DT model by 96.01% in MAE, surpasses the LightGBM model by 94.48%, and exceeds the ANFIS model by 95.68%, indicating the robust performance of ANFIS to forecast the CS of GrNCC.
3. The SHAP analysis provided that the thickness of GrN plays a pivotal role in GrNCC, significantly influencing CS and consequently exhibiting the highest SHAP value of + 9.39 for CS. The diameter of GrN, curing age, and w/c ratio are also prominent features in estimating the CS of GrNCC. The remaining feature has the least significant SHAP values. In additon, GUI can help researchers/practicioners to forcast concrete properties in due time.

The dataset used in this study contains limited data points. Therefore, this study underscores the necessity for a more extensive dataset to enhance the precision and generalizability of machine learning models. While employing interpretability through SHAP, future endeavours should explore other interpretation techniques like ICE, LIME, and PDP. Additionally, it is proposed that evolutionary algorithms be developed to improve prediction accuracy further. Furthermore, conducting a comparative assessment of graphene and its potential alternatives through a blend of experimental investigation and ML-based modelling analyses is recommended.

## Data Availability

The datasets generated or analyzed during the current study are available from the corresponding author upon reasonable request.

## Appendix A

**Table:**

S.No	SC	GD	GT	GC	WC	US	CA	CS
1	0	6	2.5	0.1	0.4	0	1	21.4
2	0	6	2.5	0.1	0.4	0	3	35.0
3	0	6	2.5	0.1	0.4	0	7	38.5
4	0	6	2.5	0.1	0.4	0	14	48.1
5	0	6	2.5	0.1	0.4	0	28	51.9
6	0	6	2.5	0.15	0.4	0	1	21.7
7	0	6	2.5	0.15	0.4	0	3	36.8
8	0	6	2.5	0.15	0.4	0	7	39.5
9	0	6	2.5	0.15	0.4	0	14	49.4
10	0	6	2.5	0.15	0.4	0	28	53.3
11	0	6	2.5	0.2	0.4	0	1	22.2
12	0	6	2.5	0.2	0.4	0	3	40.1
13	0	6	2.5	0.2	0.4	0	7	46.0
14	0	6	2.5	0.2	0.4	0	14	51.1
15	0	6	2.5	0.2	0.4	0	28	54.9
16	0	0.52	1	0.03	0.5	0	3	32.9
17	0	0.52	1	0.03	0.5	0	7	46.9
18	0	0.52	1	0.03	0.5	0	28	62.6
19	3	0.26	8	0.01	0.37	0	3	41.2
20	3	0.26	8	0.02	0.37	0	3	48.3
21	3	0.26	8	0.03	0.37	0	3	53.3
22	3	0.26	8	0.04	0.37	0	3	56.4
23	3	0.26	8	0.05	0.37	0	3	58.5
24	3	0.26	8	0.01	0.37	0	28	67.2
25	3	0.26	8	0.02	0.37	0	28	75.7
26	3	0.26	8	0.03	0.37	0	28	82.5
27	3	0.26	8	0.04	0.37	0	28	84.4
28	3	0.26	8	0.05	0.37	0	28	87.7
29	3	3.5	1	0.025	0.5	0.5	28	43.3
30	3	3.5	1	0.05	0.5	0.5	28	48.2
31	3	3.5	1	0.1	0.5	0.5	28	53.9
32	3	3.5	1	0.2	0.5	0.5	28	56.0
33	3	3.5	1	0.4	0.5	0.5	28	55.0
34	3	3.5	1	0.8	0.5	0.5	28	50.0
35	3	3.5	1	1.6	0.5	0.5	28	59.0
36	3	3.5	1	3.2	0.5	0.5	28	58.0
37	3	3.5	1	6.4	0.5	0.5	28	41.7
38	3	3.5	1	0.025	0.6	0.5	28	40.8
39	3	3.5	1	0.05	0.6	0.5	28	39.4
40	3	3.5	1	0.1	0.6	0.5	28	48.4
41	3	3.5	1	0.2	0.6	0.5	28	36.6
42	3	3.5	1	0.4	0.6	0.5	28	38.9
43	3	3.5	1	0.8	0.6	0.5	28	45.9
44	3	3.5	1	1.6	0.6	0.5	28	52.4
45	3	3.5	1	3.2	0.6	0.5	28	46.6
46	3	3.5	1	6.4	0.72	0.5	28	28.6
47	3	3.5	1	0.025	0.7	0.5	28	29.0
48	3	3.5	1	0.05	0.7	0.5	28	36.9
49	3	3.5	1	0.1	0.7	0.5	28	38.1
50	3	3.5	1	0.2	0.7	0.5	28	27.3
51	3	3.5	1	0.4	0.7	0.5	28	29.9
52	3	3.5	1	0.8	0.7	0.5	28	31.0
53	3	3.5	1	1.6	0.7	0.5	28	37.7
54	3	3.5	1	3.2	0.7	0.5	28	31.4
55	3	3.5	1	6.4	0.7	0.5	28	24.5
56	0	0.43	27.6	0.01	0.3	0	28	65.2
57	0	0.43	27.6	0.02	0.3	0	28	68.3
58	0	0.43	27.6	0.03	0.3	0	28	71.2
59	0	0.43	27.6	0.04	0.3	0	28	74.5
60	0	0.43	27.6	0.05	0.3	0	28	75.6
61	0	0.43	27.6	0.06	0.3	0	28	76.8
62	0	0.18	9.5	0.01	0.3	0	28	66.5
63	0	0.18	9.5	0.02	0.3	0	28	72.5
64	0	0.18	9.5	0.03	0.3	0	28	76.3
65	0	0.18	9.5	0.04	0.3	0	28	79.7
66	0	0.18	9.5	0.05	0.3	0	28	79.1
67	0	0.18	9.5	0.06	0.3	0	28	79.9
68	0	0.072	3.1	0.01	0.3	0	28	67.5
69	0	0.072	3.1	0.02	0.3	0	28	76.5
70	0	0.072	3.1	0.03	0.3	0	28	79.6
71	0	0.072	3.1	0.04	0.3	0	28	81.6
72	0	0.072	3.1	0.05	0.3	0	28	81.9
73	0	0.072	3.1	0.06	0.3	0	28	82.0
74	0	0.55	0.7	0.01	0.37	0	3	16.8
75	0	0.55	0.7	0.01	0.37	0	7	21.1
76	0	0.55	0.7	0.01	0.37	0	28	24.9
77	0	0.55	0.7	0.02	0.37	0	3	18.8
78	0	0.55	0.7	0.02	0.37	0	7	22.5
79	0	0.55	0.7	0.02	0.37	0	28	25.5
80	0	0.55	0.7	0.03	0.37	0	3	19.5
81	0	0.55	0.7	0.03	0.37	0	7	24.2
82	0	0.55	0.7	0.03	0.37	0	28	26.0
83	0	0.55	0.7	0.04	0.37	0	3	19.8
84	0	0.55	0.7	0.04	0.37	0	7	24.6
85	0	0.55	0.7	0.04	0.37	0	28	27.0
86	0	0.55	0.7	0.05	0.37	0	3	20.0
87	0	0.55	0.7	0.05	0.37	0	7	25.5
88	0	0.55	0.7	0.05	0.37	0	28	27.9
89	3	0.55	0.7	0.01	0.37	0	3	14.6
90	3	0.55	0.7	0.01	0.37	0	7	20.0
91	3	0.55	0.7	0.01	0.37	0	28	23.9
92	3	0.55	0.7	0.02	0.37	0	3	16.4
93	3	0.55	0.7	0.02	0.37	0	7	20.5
94	3	0.55	0.7	0.02	0.37	0	28	23.7
95	3	0.55	0.7	0.03	0.37	0	3	16.0
96	3	0.55	0.7	0.03	0.37	0	7	21.1
97	3	0.55	0.7	0.03	0.37	0	28	24.2
98	3	0.55	0.7	0.04	0.37	0	3	16.8
99	3	0.55	0.7	0.04	0.37	0	7	21.1
100	3	0.55	0.7	0.04	0.37	0	28	25.3
101	3	0.55	0.7	0.05	0.37	0	3	17.6
102	3	0.55	0.7	0.05	0.37	0	7	22.7
103	3	0.55	0.7	0.05	0.37	0	28	25.4
104	0	0.6	6	0.01	0.29	0.5	7	45.3
105	0	0.6	6	0.02	0.29	0.5	7	49.5
106	0	0.6	6	0.03	0.29	0.5	7	55.6
107	0	0.6	6	0.04	0.29	0.5	7	58.6
108	0	0.6	6	0.05	0.29	0.5	7	62.3
109	0	0.6	6	0.06	0.29	0.5	7	63.3
110	0	0.6	6	0.01	0.29	0.5	28	69.7
111	0	0.6	6	0.02	0.29	0.5	28	77.8
112	0	0.6	6	0.03	0.29	0.5	28	86.6
113	0	0.6	6	0.04	0.29	0.5	28	92.4
114	0	0.6	6	0.05	0.29	0.5	28	93.4
115	0	0.6	6	0.06	0.29	0.5	28	94.3
116	0	2	2	0.01	0.45	3	3	42.6
117	0	2	2	0.02	0.45	3	3	43.4
118	0	2	2	0.04	0.45	3	3	43.0
119	0	2	2	0.08	0.45	3	3	49.1
120	0	2	2	0.16	0.45	3	3	46.0
121	0	2	2	0.01	0.45	3	14	44.4
122	0	2	2	0.02	0.45	3	14	50.1
123	0	2	2	0.04	0.45	3	14	58.7
124	0	2	2	0.08	0.45	3	14	60.0
125	0	2	2	0.16	0.45	3	14	61.2
126	0	2	2	0.01	0.45	3	28	52.1
127	0	2	2	0.02	0.45	3	28	56.0
128	0	2	2	0.04	0.45	3	28	62.8
129	0	2	2	0.08	0.45	3	28	62.5
130	0	2	2	0.16	0.45	3	28	63.2
131	3	1.6	1	0.03	0.3	0.08	28	61.0
132	3	1.6	1	0.01	0.35	0.08	28	68.8
133	3	1.6	1	0.03	0.35	0.08	28	68.0
134	3	1.6	1	0.05	0.35	0.08	28	59.3
135	3	1.6	1	0.03	0.4	0.08	28	58.7
136	3	1.6	1	0.03	0.45	0.08	28	58.4
137	3	1.6	1	0.01	0.5	0.08	28	37.3
138	3	1.6	1	0.03	0.5	0.08	28	40.1
139	3	1.6	1	0.05	0.5	0.08	28	37.6
140	3	11.5	1.75	0.01	0.2	0.5	28	53.3
141	3	11.5	1.75	0.03	0.2	0.5	28	59.0
142	3	11.5	1.75	0.05	0.2	0.5	28	49.5
143	3	11.5	1.75	0.01	0.3	0.5	28	50.9
144	3	11.5	1.75	0.03	0.3	0.5	28	52.6
145	3	11.5	1.75	0.05	0.3	0.5	28	47.5
146	3	11.5	1.75	0.01	0.4	0.5	28	45.0
147	3	11.5	1.75	0.03	0.4	0.5	28	46.7
148	3	11.5	1.75	0.05	0.4	0.5	28	42.9
149	3	1.75	0.88	0.01	0.3	0.5	28	46.9
150	3	1.75	0.88	0.03	0.3	0.5	28	51.9
151	3	1.75	0.88	0.05	0.3	0.5	28	48.0
152	3	50	1.8	0.01	0.3	0.5	28	51.5
153	3	50	1.8	0.03	0.3	0.5	28	54.6
154	3	50	1.8	0.05	0.3	0.5	28	50.8
155	0	7.5	3.40	0.025	0.35	0.5	7	59.0
156	0	7.5	3.40	0.05	0.35	0.5	7	53.1
157	0	7.5	3.40	0.1	0.35	0.5	7	46.2
158	0	7.5	3.40	0.025	0.35	0.5	28	54.3
159	0	7.5	3.40	0.05	0.35	0.5	28	50.8
160	0	7.5	3.40	0.1	0.35	0.5	28	45.3
161	0	0.9	1	0.02	0.4	1	7	30.9
162	0	0.9	1	0.04	0.4	1	7	35.0
163	0	0.9	1	0.08	0.4	1	7	35.0
164	0	0.9	1	0.02	0.4	1	14	54.9
165	0	0.9	1	0.04	0.4	1	14	55.4
166	0	0.9	1	0.08	0.4	1	14	50.1
167	0	0.9	1	0.02	0.4	1	28	69.0
168	0	0.9	1	0.04	0.4	1	28	70.0
169	0	0.9	1	0.08	0.4	1	28	60.1
170	3	3.4	3.40	0.08	0.35	0.17	3	36.5
171	3	3.4	3.40	0.08	0.35	0.17	7	47.6
172	3	3.4	3.40	0.08	0.35	0.17	28	63.3
